# Nanostructured Microparticles Repolarize Macrophages and Induce Cell Death in an In Vitro Model of Tumour-Associated Macrophages

**DOI:** 10.3390/pharmaceutics15071895

**Published:** 2023-07-05

**Authors:** Salma Al-Fityan, Britta Diesel, Thorben Fischer, Emmanuel Ampofo, Annika Schomisch, Vida Mashayekhi, Marc Schneider, Alexandra K. Kiemer

**Affiliations:** 1 Department of Pharmacy, Pharmaceutical Biology, Saarland University, 66123 Saarbruecken, Germany; 2 Department of Pharmacy, Biopharmaceutics and Pharmaceutical Technology, Saarland University, 66123 Saarbruecken, Germanymarc.schneider@mx.uni-saarland.de (M.S.); 3 Institute for Clinical & Experimental Surgery, Saarland University, 66421 Homburg/Saar, Germany

**Keywords:** lung cancer, targeted delivery, phagocytosis, toll-like receptor, poly(I:C), inflammasome, THP-1

## Abstract

Macrophages (MΦs) in their pro-inflammatory state (M1) suppress tumour growth, while tumour-associated MΦs (TAMs) can promote tumour progression. The aim of this study was to test the hypothesis that targeted delivery of the immune activator poly(I:C) in aspherical silica microrods (µRs) can repolarize TAMs into M1-like cells. µRs (10 µm × 3 µm) were manufactured from silica nanoparticles and stabilized with dextran sulphate and polyethyleneimine. The THP-1 cell line, differentiated into MΦs, and primary human monocyte-derived MΦs (HMDMs) were treated with tumour-cell-conditioned medium (A549), but only HMDMs could be polarized towards TAMs. Flow cytometry and microscopy revealed elevated uptake of µRs by TAMs compared to non-polarized HMDMs. Flow cytometry and qPCR studies on polarization markers showed desirable effects of poly(I:C)-loaded MPs towards an M1 polarization. However, unloaded µRs also showed distinct actions, which were not induced by bacterial contaminations. Reporter cell assays showed that µRs induce the secretion of the inflammatory cytokine IL-1β. Macrophages from *Nlrp3* knockout mice showed that µRs in concentrations as low as 0.5 µR per cell can activate the inflammasome and induce cell death. In conclusion, our data show that µRs, even if unloaded, can induce inflammasome activation and cell death in low concentrations.

## 1. Introduction

Lung cancer is the leading cause of cancer-related mortality worldwide (World Health Organization, Global Cancer Observatory, 2020). Because of their prominent abundancy and their role in the tumour microenvironment, tumour-associated macrophages (TAMs) are discussed as a promising target for novel anti-tumour therapies [1,2,3]. Macrophages (MΦs) are a cell type with high plasticity [4,5], with TAMs resembling a more anti-inflammatory, M2-like polarization state, promoting tumour growth and metastasis [6,7]. In Non–Small Cell Lung Carcinoma (NSCLC), M2 TAMs are predominant over the pro-inflammatory M1-like type [8]. There exist two main strategies for targeting TAMs: One approach is to inhibit TAM abundance by killing them or inhibiting their recruitment. A second promising procedure is to reprogram TAMs to activate their anti-tumour functions [9].

Respective therapeutic approaches have to be tested in suitable in vitro TAM models. Recently, we showed that polarization of human monocyte-derived macrophages (HMDMs) with tumour-cell-conditioned medium (TCM) results in MΦs that show a high similarity to human TAMs derived from human lung cancer patients [10].

Toll-like receptors (TLRs) are members of innate immunity pattern recognition receptors (PRRs) that, upon activation, stimulate MΦs and activate an M1-like functional polarization [9]. A promising approach to revert an M2-like macrophage polarization is stimulation of the endosomal TLR3 with polyinosinic:polycytidylic acid (poly(I:C)), a synthetic double-stranded (ds)RNA, as shown in a murine model by subcutaneous injection [11]. The clinical application of poly(I:C) as an adjuvant has been limited due to heterogeneous molecular size, inconsistent activity, poor stability, and toxicity [12]. These limitations may be avoided by improving delivery and specificity: in vitro, poly(I:C) activates human alveolar macrophages and its activity can be greatly enhanced by intracellular delivery [13].

Aspherical nanostructured cylindrical silica microparticles (µRs) are a promising, inhalable drug delivery system targeting specifically MΦs. Recently, we engineered µRs loaded with plasmid DNA, showing their uptake and in vivo reporter gene expression in murine alveolar MΦs administered by nasal instillation [14]. In vitro, we showed successful delivery of siRNA against TNF to macrophage-like THP-1 cells resulting in anti-inflammatory effects [15]. In contrast, the A549 alveolar epithelial-like cancer cell line did not take up the intact particles [16]. Degradation products were taken up independent from cell type starting after 3–6 h. After 48 h, many µRs were disintegrated into nanoparticles.

With lung cancer macrophages being addressable with inhalable particulate systems [17], the aim of this work was to employ poly(I:C)-loaded µRs as drug delivery system to specifically target and repolarize TAMs by exploiting their phagocytic ability. We showed limited suitability of the frequently used THP-1 cell line to model TAMs and therefore undertook the biological characterization of µR actions in primary human TAM-like MΦs. We observed the desired repolarization of these TAM-like MΦs. Interestingly, though, unloaded µRs also showed a measurable inflammatory activation of primary MΦs and cytotoxicity even at low concentrations.

## 2. Materials and Methods

### 2.1. Materials

Cell culture media (RPMI 1640; DMEM, D6546), fetal calf serum (FCS, F7524), penicillin/streptomycin (P433), glutamine (G7513), MTT (3-[4,5-dimethylthiazol-2-yl]-2,5-diphenyltetrazolium bromide; M5655), DMSO (dimethyl sulfoxide; D8418-100 mL), crystal violet (C0775-25 G), D-PBS (D8537-500 ML), and accutase (A6964) were obtained from Sigma-Aldrich (Darmstadt, Germany). LPS (lipopolysaccharide; tlrl-peklps), FSL-1 (tlrl-fsl), Pam_3_CSK_4_ (tlrl-pms) were obtained from InvivoGen (Toulouse, France). All other materials were purchased from Sigma-Aldrich or Carl Roth (Karlsruhe, Germany) if not mentioned otherwise.

### 2.2. Cell Culture

A549 (ATCC), THP-1 (ATCC), HMDMs, and BMMs were cultured in standard growth medium (RPMI 1640, 10% FCS, 100 U/mL penicillin G, 100 μg/mL streptomycin, 2 mM glutamine). All cell lines were maintained at 37 °C and 5% CO_2_.

#### 2.2.1. THP-1

THP-1 cells were seeded in a 12-well plate at a density of 250,000 cells/well. Cells were differentiated into a macrophage-like phenotype by incubation with 100 nM phorbol-12-myristate-13-acetate (PMA, #524400, Sigma-Aldrich) for 48 h. To generate TAM-like THP-1 macrophages, cells were incubated with tumour cell conditioned medium (TCM) for 24 h. TCM was collected from a confluent layer of A549 cells after 48 h, filtered (0.22 µm), and used for polarization for 24 h.

#### 2.2.2. Primary Peripheral Blood Human Monocyte-Derived Macrophages (HMDMs)

Monocytes were isolated from buffy coats of healthy adult blood donors (Blood Donation Center, Klinikum Saarbrücken, Germany). The local Ethics Committee approval (permission no. 173/18, State Medical Board of Registration, Saarland, Germany) was obtained. HMDM isolation was performed as described previously [18,19]. In brief, peripheral blood mononuclear cells (PBMCs) were isolated from buffy coats by density gradient centrifugation using Lymphocyte Separation Medium 1077 (C-44010, PromoCell, Heidelberg, Germany) and LeucoSEP tubes (227290, Greiner Bio-One, Kremsmünster, Austria). Monocytes were purified by magnetic cell sorting using anti-CD14 microbeads (130-050-201, Miltenyi Biotec, Bergisch Gladbach, Germany) and LS Columns (130-042-401, Miltenyi Biotec) according to the manufacturers’ instructions, except that only 10% of the recommended bead amount was used [19]. Monocyte purity was >95% as assessed by CD14 expression.

The obtained monocytes were seeded as indicated in Table 1 and differentiated into macrophages in standard growth medium supplemented with 20 ng/mL human macrophage-colony stimulating factor (M-CSF, Miltenyi Biotec, 130-096-492) at 37 °C and 5% CO_2_ for 5 days. After differentiation, macrophages were left without polarization (M0) or polarized towards M1 by 20 ng/mL IFN-γ (130-096-484, Miltenyi Biotec) and 100 ng/mL LPS for 24 h. TAM-like macrophages were incubated in tumour cell-conditioned medium (TCM) supplemented with 20 ng/mL M-CSF.

#### 2.2.3. Reporter Cell Lines

HEK-Dual™ hTLR2 (hkd-htlr2ni), HEK-Blue™-hTLR2 (hkb-htlr2), HEK-Blue™ IL-1R (hkb-il1r), and THP1-XBlue™ (thpx-sp) reporter cells and selection antibiotics were obtained from InvivoGen and maintained after the supplier’s suggestion. HEK-Dual™ hTLR2, HEK-Blue™-hTLR2, and HEK-Blue™ IL-1R reporter cells were grown in DMEM with 10% heat-inactivated FCS (30 min at 65 °C), 2 mM glutamine, 50 U/mL penicillin G, 100 μg/mL Normocin and selection antibiotics (100 μg/mL of Hygromycin B Gold and 50 μg/mL of Zeocin for HEK-Dual™ hTLR2; 1× HEK-Blue™ Selection for HEK-Blue™-hTLR2; 200 µg/mL Hygromycin B Gold, 1 µg/mL Puromycin and Zeocin for HEK-Blue™ IL-1R). THP1-XBlue™ were maintained in RPMI 1640 with heat-inactivated (30 min at 65 °C) 10% FCS, 2 mM glutamine, 50 U/mL penicillin G, 50 μg/mL streptomycin, and 200 µg/mL Zeocin. The measurement medium did not contain Normocin and selection antibiotics as described previously [10].

#### 2.2.4. Primary Murine Bone Marrow-Derived Macrophages (BMMs)

Mice were held in a 12/12-h light/dark cycle with food and water ad libitum. BMMs were obtained from C57BL/6 wild type (WT) or NLRP3 knockout (KO) mice (The Jackson Laboratory, approval number 2.4.2.2.-06/2020). The cells were isolated according to previously described methods [20]. Briefly, femurs and tibias were flushed with standard growth medium. Erythrocytes were lysed with hypotonic buffer after centrifuging. Obtained cells were cultured overnight (~16 h) in medium supplemented with mouse M-CSF (130-101-704, Miltenyi Biotec) in a 150-cm^2^ flask. The next day, non-adherent cells were collected and cultivated for 5 days in a 150-cm^2^ flask. On day 6, cells were detached with accutase and seeded in 96-well plates for further analysis (20,000 cells/well).

### 2.3. Particle Synthesis and Characterization

#### 2.3.1. Synthesis of Aspherical Cylindrical Silica Microparticles (µRs)

Cylindric-shaped microparticles (µRs; 10 µm × 3 µm) were manufactured as described previously employing a track-etched polycarbonate filter membrane [14,21]. Briefly, non-fluorescent or fluorescent amorphous silica beads (PSI-0.02; PSI-G0.2, Kisker Biotech, Steinfurt, Germany) were assembled to µRs and stabilized with three polymer double-layers from branched polyethyleneimine 25 kDa (PEI, Sigma-Aldrich) and dextran sulphate 10 HS 10 kDa (TdB Labs, Uppsala, Sweden) [22,23]. The layer-by layer technology introduced by Decher et al. [24] combining oppositely charged polymers was shown to work very well also for stabilizing the rod-shaped nanoparticle assembly [19]. Poly(I:C)-loaded µRs (polyinosinic:polycytidylic acid; tlrl-pic; high molecular weight, InvivoGen) were manufactured similarly, as described in [15,25] exchanging a negatively charged polymeric layer by poly(I:C). Mass median aerodynamic diameter MMAD = 2.53 ± 0.23 µm and Fine Particle Fraction FPF = 34 ± 5% were determined as described before [26,27]. Shape and homogeneity of the particles are depicted in the SEM and CLSM micrographs shown in Figure S5.

µRs were suspended by sonication and vortexing at a concentration of 50 µg/µL in D-PBS or water. They were used immediately or stored at −20 °C. After thawing, µRs were again sonicated and vortexed to archive a homogeneously dispersed suspension. On average, 1 mg µRs contained 2.5 million µRs. For some experiments, µRs were counted in a Neubauer counting chamber employing an Axiovert 40 CFL Microscope (Carl Zeiss™, Jena, Germany) or by using a LUNA-FL™ Dual Fluorescence Cell Counter (Logos Biosystems, Villeneuve d’Ascq, France). Before application, µRs were vortexed carefully. After treatment, cell culture plates were centrifuged with 100× *g* for 30 s. For disintegration, µRs were diluted in RPMI 1640 supplemented with 100 U/mL penicillin and 100 μg/mL streptomycin and stored at 37 °C for 4 days under sterile conditions. See Table 2 for conversion from µR concentration to treatments (if not counted).

#### 2.3.2. Poly(I:C)-Release from µRs

A release study was performed in a salt mixture resembling a phagolysosome’s physiological condition following Stefaniak et al. [28] as described previously [15]. In brief, µRs were incubated in 0.5 mL salt solution at 37 °C under shaking conditions for the indicated period. The solution was centrifuged at 20,000× *g* for 15 min, supernatant was mixed with SYBR^®^ Gold (S11494, Invitrogen, Waltham, MA, USA), and the fluorescence measured at 485 nm.

### 2.4. Analysis of Cellular µR Uptake

#### 2.4.1. Flow Cytometry

Phagocytosis was determined in principle as described previously [13]. After treatment, MΦs were washed five times with PBS, and a preheated (37 °C) solution of 0.5 µM CellTracker™ Deep Red Dye (C34565, ThermoFisher Scientific, Munich, Germany) in an FCS-free medium was added. After 30 min in an incubator, cells were washed with ice-cold PBS. The cells were detached in ice-cold PBS, fixed in ice-cold 1% paraformaldehyde solution, stored on ice, and analysed on a BD LSRFortessa™BD (Biosciences, San Jose, CA, USA) with BD FACSDiva™ software (v8.0.1). Results were additionally analysed using the BD FACSuite^TM^ software (v1.0.6).

#### 2.4.2. Live-Cell Microscopy-Based Analysis

HMDMs were stained with a preheated (37 °C) solution of 0.5 µM CellTracker™ Deep Red Dye in an FCS-free medium. After 30 min, the supernatant was removed, and cells were polarized for 48 h, as described above, before treatment. Cells were analysed for 1 h with an IncuCyte^®^ S3 live-cell analysis system, in principle as described previously (Essen BioScience, Royston, UK [29]). Phase-contrast and fluorescent scans were taken every 10 min with a 20× objective. The number of µR-positive HMDMs was determined as HMDMs with green- and red-positive fluorescence signals per time point employing the IncuCyte^®^ Cell-By-Cell Analysis Software Module version 2019B Rev2 (% cells/cell count).

#### 2.4.3. Confocal Laser Scanning Microscopy (CLSM)

Cells were incubated with 2 green-fluorescent µRs/cell for 20 min or kept untreated, fixed with ice-cold 4% PFA solution and permeabilized in 0.25% triton X-100 solution as described [30,31]. Blocking was with 1% BSA solution before staining with 0.766 µM Phalloidin–Tetramethylrhodamine B isothiocyanate (P1951, Sigma-Aldrich; in 1% BSA). Cells were stained with 5 µg/mL DAPI (4′,6-Diamidino-2-phenylindole dihydrochloride, D9542-1 MG, Sigma-Aldrich) and fixed with FluorSave™ (345789, Calbiochem, Merck, Darmstadt, Germany). Slides were analysed employing a Confocal Laser Scanning Microscope (CLSM; LSM 510 Meta, Zeiss). The images were analysed, edited, and exported using Zen 3.0 software (blue edition; Zeiss).

### 2.5. Surface Protein Expression Analysis Employing Flow Cytometry

Flow cytometric analysis was performed as described previously [10,32]. Briefly, after 48 h of treatments, HMDMs were washed, detached in 2.5 mM EDTA solution on ice and blocked with BD Fc Block™Pure (564220, BD Biosciences, San Jose, CA, USA) for 10 min at room temperature. HLA-DRPerCP-Cy5.5 (552764; Clone: G46-6, BD Biosciences), CD80 BB51 (565008; Clone: L207.4, BD Biosciences), CD163PE-CF594 (562670; Clone: GHI/61, BD Biosciences) or CD14 APC (555399; Clone: M5E2, BD Biosciences) antibodies were added in each sample and incubated on ice for 30 min according to manufacturers’ suggestions. HMDMs were washed and fixed with ice-cold 1% PFA solution, stored on ice, and analysed with a BD LSRFortessa™ Cell Analyzer and the BD FACSDiva v8.0.1 software (BD Biosciences). To quantify the surface marker expression, median fluorescence intensities of singlet cells were used.

### 2.6. RNA Isolation, Reverse Transcription, and Quantitative RT-PCR (qPCR)

Total RNA was isolated from HMDMs as described previously [10,13] employing the High Pure RNA Isolation Kit (11828665001; Roche Diagnostics International, Rotkreuz, Switzerland), and the High-Capacity cDNA Reverse Transcription Kit (4368813; Applied Biosystems, Foster City, CA, USA) according to the manufacturers’ instructions. qPCR was performed employing a CFX96 Touch™ Real-Time PCR detection system (Bio-Rad, Hercules, CA, USA). The reaction protocol was 95 °C for 15 s, followed by 40 cycles of 94 °C for 20 s, 60 or 61 °C for 20 s, and 72 °C for 20 s. Then, 5× Hot FirePOl EvaGreen qPCR Mix (08-25-00020; Solis BioDyne, Tartu, Estonia) was employed for gene expression analysis with primers as shown in Table 3.

Data were analysed by absolute quantification using a standard curve of the PCR product cloned into the pGEM-T Easy vector (Promega, Walldorf, Germany). All samples and standards were analysed in triplicates and melting curve analysis was performed as a quality control. Data were normalized to the housekeeping gene *18S*.

### 2.7. Endotoxin Assay

For the detection of potential endotoxin contamination, the PyroGene^TM^ Recombinant Factor C Endpoint Fluorescent Assay (50-658U; Lonza, Basel, Switzerland) with a detection limit of 0.05–0.005 EU/mL was employed as described in the manufacturers’ instructions. Nine different µR batches were analysed in concentrations as used for treatments (100 and 200 µg/mL) in three different experiments. Because µRs are expected to disintegrate within hours in culture media [16], disintegrated µRs were also analysed. In each experiment, spike controls with exogenous LPS (0.05 EU/mL) were included (accepted recovery rate 50–200% as recommended by the manufacturer).

### 2.8. Reporter Cell Assay (HEK-Dual™ hTLR2, HEK-Blue™-hTLR2, HEK-Blue™ IL-1R and THP1-XBlue™)

Reporter cells from InvivoGen express a set of receptors as indicated in Table 4. An NF-κB/AP1-inducible secreted embryonic alkaline phosphatase (SEAP) reporter gene was employed as an indicator for inflammatory activation according to the manufacturers’ instructions and as described [33].

HEK-Dual™ hTLR2, HEK-Blue™-hTLR2 (5 × 10^5^ cells/well), and THP1-XBlue™ (10 × 10^5^ cells/well) cells were seeded into 96-well plates and immediately treated as indicated to monitor receptor-dependent activation. After 24 h, 20 µL supernatant from each well was incubated with 180 µL Quanti-Blue™ solution (SEAP detection medium; rep-qbs; InvivoGen) for 6 h.

HEK-Blue™ IL-1R reporter cells were seeded into 96-well plates (5 × 10^5^ cells/well) in 180 µL cell culture medium and incubated with 20 µL supernatant of HMDM or BMM treated with µRs/samples for 24 h. Control supernatants (LPS followed by MSU or ATP) of BMM cells were diluted 1:100 before adding. To determine IL-1β secretion, 20 µL of the reporter cell supernatants were incubated with 180 µL Quanti-Blue™ solution for 3 h. Human or murine IL-1β (rcyec-hil1b, InvivoGen; 130-101-681, Miltenyi Biotec) was employed for the standard curve.

SEAP activity was determined at 600 nm in a microplate reader (GloMax^®^ Discover Microplate Reader, Promega). Cell viability was determined via MTT assay after removal of supernatants (Figures S3 and S4). IL-1B concentration (pg/mL) was calculated with a four-parameter logistic curve fit (Myassays.com (accessed on 17 August 2021)).

### 2.9. Determination of Cell Viability

#### 2.9.1. MTT Assay

The MTT [3-(4,5-dimethyl-thiazol-2-)-2.5-diphenyl tetrazolium bromide] colorimetric assay was used with 50,000 cells/well in 96-well plates as described previously [18]. In brief, after 24 h of cell treatment, the medium was removed, and MTT solution (0.5 mg/mL in medium) was added. After 20–60 min the MTT solution was removed, and the formazan crystals were solubilized with DMSO. Absorbance was measured at 560 nm in a GloMax^®^ Discover Microplate Reader (Promega). Values for each treated well were normalized to the untreated control. In order to evaluate a possible interaction of the (nano-) materials measured, every step was controlled by addition of µRs (e.g., addition to MTT solution, to DMSO, to cells without adding MTT).

#### 2.9.2. Live-Cell Microscopy-Based Analysis

Cytotoxicity was assessed as described previously with an IncuCyte^®^ S3 live-cell analysis system [3]. In brief, after cell differentiation into HMDMs, cells were incubated as indicated with µRs, controls and 250 nM IncuCyte^®^ Cytotox Red Reagent (4632, Sartorius, Göttingen, Germany). Phase-contrast and fluorescent scans were taken every 2 h with a 20× objective for an additional 4 h. Cell viability is shown as IncuCyte^®^ Cytotox Red Reagent negative cells per treatment in % of cell count normalized to untreated control using the add-on IncuCyte^®^ Cell-By-Cell Analysis Software Module (version 2019B Rev2).

### 2.10. Statistics

Data are shown as means ± SEM (standard error of the mean) in column and line charts or box charts as 25th/75th percentile boxes, geometric medians, means (square), measurement points (rhomb), and 1.5 interquartile range (whiskers) unless stated otherwise ± SD (standard deviation). To determine *p*-values, ANOVA with post hoc Bonferroni correction for normally distributed data or Mann–Whitney U test with Bonferroni correction for not normally distributed data were employed. In addition, the Grubbs’ test was utilized to identify outliers. The OriginPro 2019 software (OriginLab, Northampton, MA, USA) was used for statistical analyses and illustrations. 

## 3. Results

### 3.1. Uptake of Rod-Shaped Microparticles by TAMs

**Figure 1:**
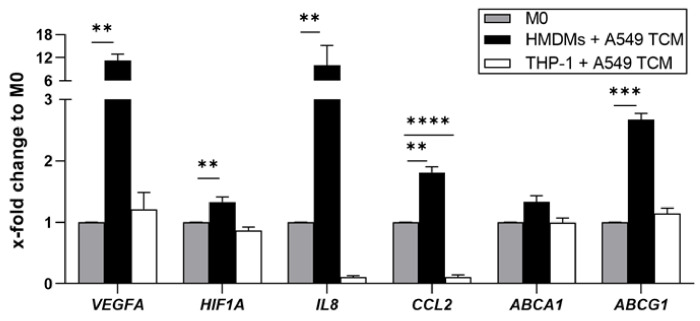
Expression profile of THP-1 MΦs and HMDMs polarized with TCM towards TAM-like MΦs. VEGFA, HIF1A, IL8, CCL2, ABCA1 and ABCG1 mRNA expression as determined by qPCR (HMDMs: *n* = 3 individual donors; THP-1: *n* = 2; triplicates). ** *p* < 0.01, *** *p* < 0.001, **** *p* < 0.0001.

Rod-shaped nanostructured microparticles (microrods, µRs) have been shown as an interesting approach to target macrophages [15]. This previous work was carried out in THP-1 macrophages, which represent a frequently used and well-established model for human macrophages when studying inflammatory cell activation [34]. In order to test whether THP-1 macrophages are also suitable to model human TAMs, we compared their response towards treatment with TCM derived from A549 lung carcinoma cells. Surprisingly, regarding gene expression of a number of genes known to be increased both in ex vivo human TAMs as well as in in vitro HMDM-derived TAM-like macrophages [10], THP-1 macrophages either did not show any expression change (*VEGFA*, *HIF1A*, *ABCA1*, *ABCG1*) or the expression was reduced instead of increased (*IL8*, *CCL2*; Figure 1). Accordingly, we decided to use TAMs polarized from primary HMDMs (=TAMs) instead of THP-1 macrophages to characterize the suitability of µRs to repolarize TAMs.

**Figure 2:**
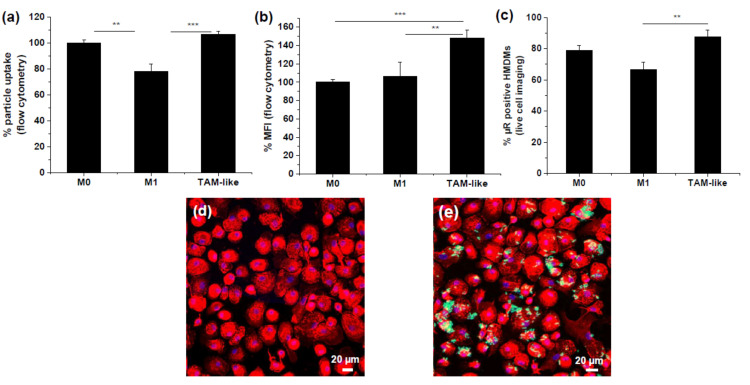
Rapid particle uptake by primary human macrophages. For flow cytometric analysis (**a**,**b**), HMDMs (M0, M1, and TAMs) were incubated for 20 min with green fluorescent µRs (2 µRs/cell, FITC) and stained with CellTracker ™ Deep Red Dye (APC). (**a**) Mean of normalized FITC MFI (median fluorescence intensity) of M1 and TAMs relative to M0 (100%) of CellTracker ™ positive events. (**b**) Percentage of FITC-positive cells in CellTracker™ positive HMDMs normalized to FITC positive M0 (100%). (*n* = 5, duplicates). (**c**) IncuCyte^®^ live-cell microscopy-based analysis of mean of µR-positive (high green) and CellTracker™ positive (high red) HMDMs in % of cell count after 20 min (1 µR/cell, *n* = 4 individual donors, triplicates). (**d**,**e**) µR uptake by M0 HMDMs as determined with CLSM. Untreated HMDMs (**d**) or after incubation with µRs (**e**) (20 min, 2 µR/cell). Red: F-actin stained with Phalloidin–Tetramethylrhodamine B isothiocyanate, blue: Nucleus stained with DAPI, green: µRs. Representative images (*n* = 3, duplicates). ** *p* < 0.01, *** *p* < 0.001.

Uptake of µRs by HMDMs was quantified by flow cytometry (Figure S1 and Figure 2). In order to assess whether different polarization states take up µRs to a different extent, we compared the uptake by TAMs to non-polarized M0 macrophages and inflammatory M1 macrophages (polarized by treatment with LPS/IFNγ). A higher proportion of TAMs and M0 took up µRs than M1 MΦs (Figure 2a). The µR-positive TAMs took up distinctly more µRs than M0 and M1 MΦs (Figure 2b). Uptake was confirmed by CLSM (Figure 2d,e).

### 3.2. Loaded and Unloaded µRs Lead to an M1-Like Phenotype

**Figure 3:**
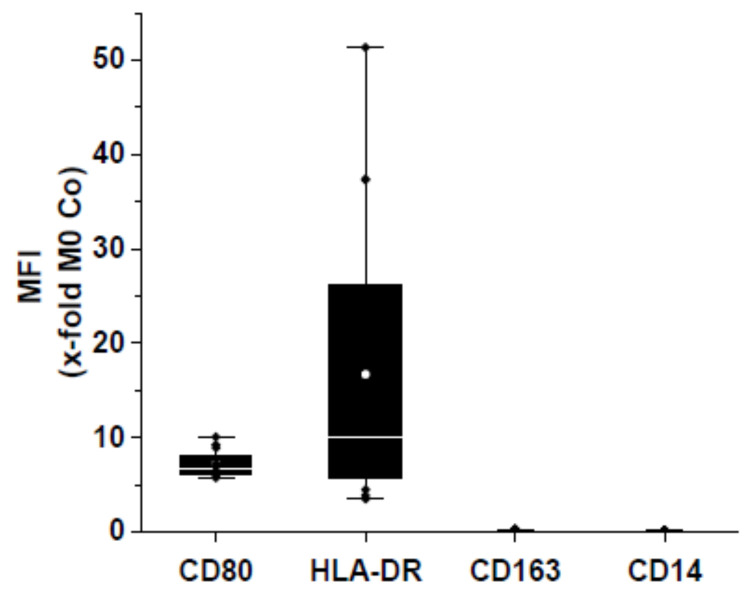
Characterization of MΦs polarized towards the M1-like phenotype by 24 h of LPS/IFNγ. Surface markers CD80, HLA-DR, CD163, and CD14 were determined by flow cytometry. Mean of normalized FITC MFI (median fluorescence intensity) of M1 relative to M0 (100%; *n* = 5, duplicates).

In order to assess a polarization towards an M1-like phenotype we employed a classical M1 polarization of HMDMs by the treatment with LPS and IFNγ. As read-out parameters for an M1 polarization we confirmed an altered composition of surface markers as well as an altered gene expression: M1 macrophages exhibited elevated amounts of CD80 and HLA-DR and lower amounts of CD14 und CD163 on their surface (Figure 3). The same donors were employed to assess µR action.

**Figure 4:**
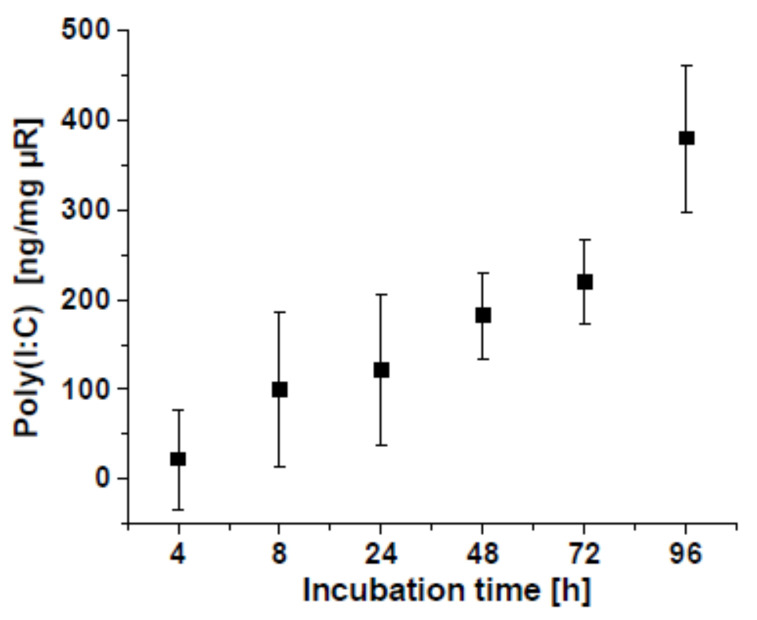
Poly(I:C)-release from µRs in phagolysosomal simulant fluid. µRs were gently shaken at 37 °C for the indicated time and centrifuged at 20,000× *g* for 15 min. Supernatants were mixed with SYBR^®^ Gold. Fluorescence ± SD at 485 nm (triplicates).

**Figure 5:**
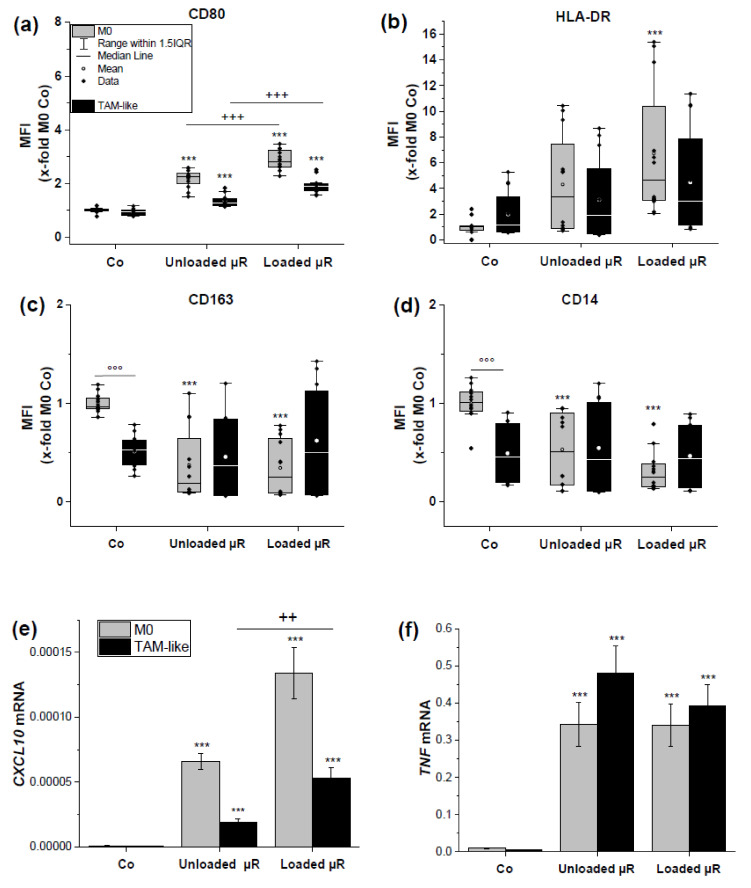
µR treatment increases M1 surface marker and *CXCL10* and *TNF* mRNA expression in TAMs. Expression of surface markers (**a**) CD80, (**b**) HLA-DR, (**c**) CD163, and (**d**) CD14 in M0 HMDMs, or TAMs as determined by flow cytometry. Incubation with 0.5 non-fluorescent µR/cell poly(I:C)-loaded µRs (=loaded µRs), or unloaded µRs. MFI x-fold of untreated M0 HMDM; *n* = 4, triplicates. *CXCL10* (**e**) and *TNF* mRNA (**f**) expression relative to *18S* in M0 HMDMs (grey) and TAMs (black) were untreated (Co) or incubated with unloaded or loaded 0.5 µR/cell. (*n* = 3, individual donors, triplicates). Treatment vs. untreated: *** *p* < 0.001; loaded µR vs. unloaded: ^++^ *p* < 0.01, ^+++^ *p* < 0.001, TAM vs. M0: °°° *p* < 0.001.

Both M0 as well as TAMs were treated either with unloaded µRs or with µRs loaded with the TLR3 agonist poly(I:C), with a poly(I:C) release of 5.6 ng/mg µR/h on average (Figure 4). Loaded µRs induced a clear M1 polarization as assessed by elevated CD80 and HLA-DR and lowered CD163 and CD14 surface marker expression (gating strategy in Figure S2, results Figure 5a–d) as well as elevated *CXCL10* and *TNF* expression (Figure 5e,f). Interestingly, though, a distinct effect towards an M1 polarization was also exhibited by unloaded µRs, although to a lower extent (Figure 5). In general, the effects seemed stronger on M0 macrophages than on TAMs.

### 3.3. Evaluation of µRs’ Inflammatory Potential

We were surprised by the strong effect of unloaded µRs on macrophage polarization and hypothesized that this might be caused by a contamination with bacterial components although µRs had been produced under sterile conditions. In fact, in contrast to THP-1 macrophages [15], PBMCs are very sensitive to bacterial endotoxins. We therefore undertook a careful determination of possible µR contaminations.

Because of the high interaction potential of nano- and microparticles with the classical LAL assay [35,36,37,38], we employed a recombinant Factor C assay including spike controls. Whereas spiking with intact µR resulted in the desired range (50–200%), spiking with disintegrated rods resulted in a non-acceptable variability.

With a limit of detection of 0.005 EU/mL in the recombinant factor C assay, no endotoxin contamination in four different intact µR batches was detected in concentrations, which match the ones used in functional assays (100 and 200 µg/mL; duplicates).

**Figure 6:**
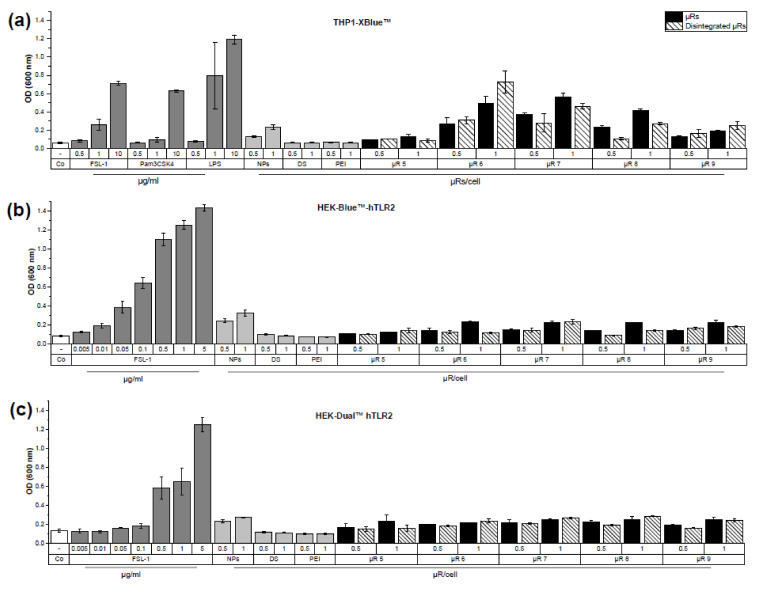
µR incubation leads to TLR activation in THP1-XBlue™ (**a**), HEK-Blue™-hTLR2 (**b**), and HEK-Dual™ hTLR2 (**c**) reporter cells. Cells were left untreated (Co) or incubated with corresponding positive controls: FSL-1, Pam_3_CSK_4_ and LPS (ng/mL); components of silica µRs in an amount which corresponds to 0.5 or 1 µR/cell. Silica nanoparticles (NPs): 100 µg/mL, 200 µg/mL; dextran sulphate (DS) and branched polyethyleneimine (PEI): 2.5 and 5 µg/mL; with 100 or 200 µg/mL µRs (0.5 and 1 µR/cell) of different production batches (µR 5–9). µRs were unchanged (black) or disintegrated (shaded); mean optical density ± SD, duplicates.

Since the activation of TLRs may be amplified if their ligands are administered as particulate structures [39], we assessed the µRs’ inflammatory potential in a panel of human and murine reporter cell lines expressing a defined subset of PRRs (Table 3, Figure 6) that were clearly activated by a panel of TLR agonists (FSL-1, LPS, and Pam_3_CSK_4_). µRs were applied to reporter cells in concentrations, which did not influence their viability (Figure S3).

µR batches activated THP1-XBlue™, HEK-Blue™-hTLR2, and HEK-Dual™ hTLR2 cells to a different extent (Figure 6). Because of the lack of activation of hTLR2 cells and no responsiveness in the recombinant factor C assay (showing absence of LPS/TLR4 activation), we could exclude that the inflammatory cell activation exhibited by µRs is facilitated due to a contamination with bacterial cell wall/membrane component agonists. To investigate whether the detected cell activation is dependent on microparticle integrity, disintegrated µRs were employed [16], but the activating potential of intact and disintegrated µRs was similar. Silica nanoparticles resulted in a slightly higher inflammatory cell activation than the other raw materials employed for µR synthesis, i.e., the coating substances dextran sulphate (DS) and branched polyethyleneimine (PEI), which showed no activity.

### 3.4. µRs Promote IL-1β Secretion in HMDMs

**Figure 7:**
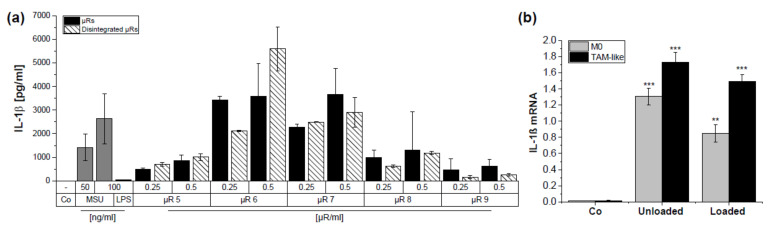
µR incubation leads to IL-1β secretion in HMDMs. (**a**): IL-1β [pg/mL] was measured in HEK-Blue™ IL-1R reporter cells after incubation with HMDM supernatant for 24 h. Cells were left untreated (Co) or incubated with positive controls (LPS (100 ng/mL) for 18 h ± monosodium urate crystals (50 and 100 µg/mL) for 6 h) or 50/100 µg/mL µRs (0.25 and 0.5 µR/cell) of different production batches (µR 5–9). µRs were unchanged or disintegrated; duplicates. IL1B mRNA (**b**) expression relative to 18S in M0 HMDMs (grey) and TAMs (black), untreated (Co) or incubated with unloaded or loaded 0.5 µR/cell. (Mean ± SD, *n* = 3, triplicates). ** *p* < 0.01, *** *p* < 0.001.

The results from the reporter cell lines suggested that the particulate nature may be responsible for the immune activating potential of µRs. Since the NLRP3 inflammasome represents a sensor of particulate matter [40], we assessed the production of IL-1β as a readout parameter for inflammasome activation (Figure 7a). The positive control monosodium urate crystals and all µR batches induced IL-1β secretion, although to a different extent. Again, there was no clear indication for an effect of µR disintegration. Accordingly, *IL-1B* mRNA expression was also induced by unloaded and loaded µR in human M0 and TAMs (Figure 7b).

### 3.5. µRs Induced IL-1β Secretion Is NLRP3 Dependent

**Figure 8:**
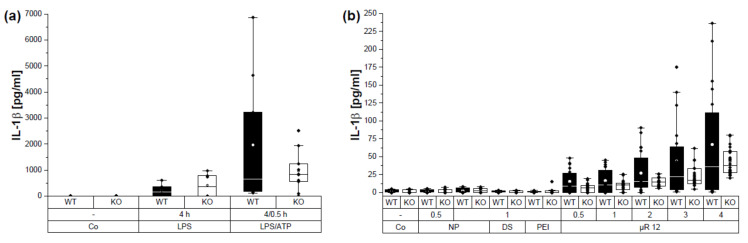
µR incubation leads to NLRP3-dependent IL-1β secretion in BMMs. IL-1β [pg/mL] was measured in HEK-Blue™ IL-1R reporter cells after incubation with supernatants from C57BL/6 WT, C57BL/6 *Nlrp3* KO for 24 h. (**a**): Cells were left untreated (Co); incubated with the positive control LPS (100 ng/mL) for 4 h, followed by ATP treatment (3 mM) for 30 min. (**b**): Co and components of silica µRs in an amount, which corresponds to 0.5 or 1 µR/cell: 100 µg/mL, 200 µg/mL; dextran sulphate (DS) and branched polyethyleneimine (PEI): 2.5 and 5 µg/mL; with 100 or 200 µg/mL µRs (0.5 and 1 µR/cell) of production batch 12 (µR 12). *n* = 2–6, mean ± SD, duplicates.

Because of the potential of unloaded µRs to induce IL-1β secretion, NLRP3 activation was evaluated, employing BMMs from WT and *Nlrp3* KO mice. The positive control, LPS treatment followed by ATP, induced a lower IL-1β secretion in *Nlrp3* KO than WT BMMs (Figure 8a). Despite a high donor dependency, IL-1β secretion in supernatants from BMMs treated with µRs was lower in *Nlrp3* KO than in WT BMMs (Figure 8b) suggesting an activation of the NLPR3 inflammasome by µRs.

### 3.6. Cytotoxicity Is Involved in µR Effect on Viability

**Figure 9:**
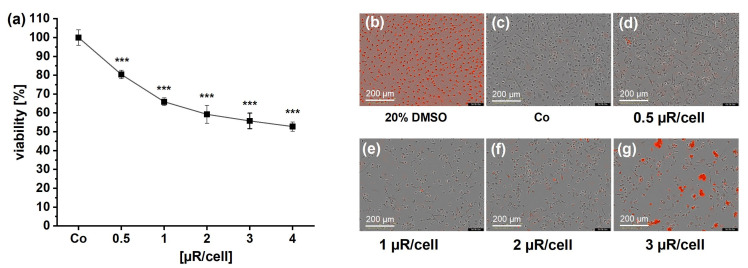
HMDM viability after incubation with µRs. (**a**): MTT assay incubated with µRs for 24 h (*n* = 3–5, mean absorption normalized to untreated controls (Co, 100%), 4 technical replicates, 4 different µR batches. (**b**–**g**) Representative images taken by IncuCyte^®^ S3 live-cell analysis system after 48 h of µR incubation and IncuCyte^®^ Cytotox Red Reagent detection (red, 20× objective). Positive control: 20% DMSO. *n* = 4, triplicates, one µR batch. *** *p* < 0.001.

Because of the possible involvement of NLRP3 in inflammatory cell activation by µRs and the fact that inflammasome activation induces cell death [31], we performed a detailed analysis of cell viability after µR treatment by measuring metabolic changes (MTT assay) and by live-cell microscopy-based analysis. In HMDMs, after 24 h of incubation time, cell viability was slightly but significantly reduced already at concentrations as low as 0.5 µR/cell, independent of the assay or the µR batch (Figure 9).

**Figure 10:**
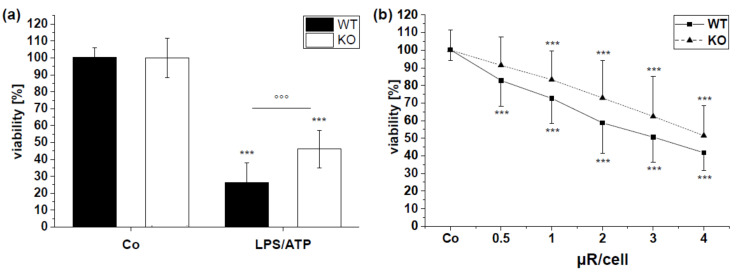
BMM wildtype and *Nlrp3* KO viability determined by MTT-assay after 24 h. BMMs were left untreated (Co; **a**,**b**), treated with positive control LPS (100 ng/mL) for 4 h, followed by ATP treatment (3 mM) for 30 min (**b**) or treated with µRs. Mean absorption (±SD) normalized to untreated controls of each genotype (Co, 100%). (**a**): WT: *n* = 2–6, duplicates, KO: *n* = 2–4, duplicates, (**b**) WT: *n* = 9, triplicates, KO: *n* = 6, triplicates. Treatment vs. untreated: *** *p* < 0.001; TAM vs. M0: °°° *p* < 0.001.

Viability of WT and *Nlrp3* KO BMMs was reduced by the positive control LPS/ATP (Figure 10a). Similar to HMDMs, there was a reduction in viability after treatment with µRs (Figure 10b). *Nlrp3* knockout BMM viability was less affected by µRs compared to WT. These data indicate a dose-dependent role of cytotoxicity of µRs, which is NLRP3-dependent.

## 4. Discussion

The µRs in this study have been used successfully employing THP-1 cells as a model for human macrophages. In this type of cells, we showed a sufficient uptake and delivery, in combination with a low cellular toxicity and a good reproducibility [15]. As we show now, the usage of THP-1 macrophages as a model for TAMs is limited because of a very different gene expression compared to relevant genes from the “gold standard”, i.e., ex vivo human TAMs [10]. In this study we confirm that the polarization of in vitro differentiated HMDMs by TCM recapitulates key features of ex vivo TAMs [10]. In addition, the finding that in human NSCLC the predominant MΦ population represents monocyte-derived macrophages [41] emphasizes the suitability of HMDMs polarized into TAMs as a cell model to characterize targeting strategies for lung cancer macrophages.

Our previous findings showed that ex vivo lung cancer TAMs take up more silica nanoparticles than alveolar macrophages from non-tumour lungs [18]. Similarly, in this study, in vitro polarized TAMs took up more µRs than non-polarized HMDMs and also the proportion of phagocytosing cells was higher. In general, a higher phagocytic activity of M2 macrophages than M1 has been described [42,43].

The µRs were originally designed for pulmonary administration, making them well suitable for trying to reach and repolarize TAMs in the lung. The particles (10 µm × 3 µm) align in the air stream making the thinner side responsible for the aerodynamic properties, which promise deposition in the deep lung (MMAD = 2.53 ± 0.23 µm, FPF = 34 ± 5%). Addressing TAMs from the air side by inhalation seems straightforward with respect to depositing the carriers to the site of action.

The dominant clearance mechanism in the deep lungs is internalization by professional phagocytes, e.g., alveolar macrophages (AMs) [44], which are, in contrast to lung epithelial or endothelial cells, the only cells which take up particles bigger than ~0.5 µm [45]. It has been shown in mice that AMs take up particles and infiltrate lung tumour margins [17]. In addition to stromal TAM density, alveolar TAM density has been described to be significantly associated with a variety of biological and clinical factors (e.g., tumour differentiation, pathological stage) as well as with a poor prognosis in NSCLC patients [46].

A close contact of µRs to TAMs may also be possible by usage as a second line of treatment after surgical cancer removal. Taking into account the current knowledge of the immunobiology of TAMs [5], it is likely that µR-based targeting of alveolar or stromal M2-like TAMs may be useful in complementing surgical therapy or immune cell-based immunotherapy. In this study, we provide further evidence for the suitability of aspherical silica microparticles for (i) specific targeting, as shown by a higher degree of µR uptake by TAMs compared to M1 MΦs and (ii) delivery of cargo to TAMs, as we show by a significant change of cell surface markers to a more M1-like phenotype for poly(I:C)-loaded µRs. In addition, the expression of inflammatory genes *TNF* and *CXCL10* was increased after treatment with poly(I:C)-loaded particles. Poly(I:C) is a promising candidate for intracellular delivery of MΦs: it is described that transfection of poly(I:C) increases the inflammatory response at least 100-fold in HMDMs compared to its addition to the cell culture medium [47]. One approach to overcome biodegradation and targeting issues of poly(I:C) are nano-delivery systems, e.g., arginine-based nano complexes were employed in vitro to target M2-like HMDMs for repolarization to an M1-like phenotype [48]. It is also being evaluated in clinical trials, e.g., in a nanocomplex formulation with polyethyleneimine [49].

It is important to note that treatment of MΦs with unloaded aspherical particles in the µm-range also resulted in a smaller, but also significant, pro-inflammatory activation of TAMs and M0 MΦs, which was not detected in THP-1-derived macrophages to a significant extent [15,25].

In contrast to epithelial or macrophage-like cell lines (A549, THP-1) [16,25], primary MΦs are very susceptible to bacterial endotoxins. The biologic activity of very low LPS concentrations was detected using intracellular TNF staining of monocytes [50]. Because of this high sensitivity of primary human MΦs, a careful analysis of µR employing the recombinant factor C assay was performed. Whereas no contamination with the TLR4 ligand LPS was observed in µR, the disintegrated µRs interacted with the assay, a problem frequently experienced with (nano)-particulate materials [37]. Because disintegration is a process needed in the cell for cargo delivery, disintegrated particles also have to be taken into account in endotoxin testing.

Therefore, we employed THP-1 reporter cells with a broad variety of PPRs, including TLR4, in addition to two cell lines detecting TLR2 agonists. The sensitivity of TLR4 reporter cells was shown to be comparable to the frequently used LAL assay [51]. The cells employed in this study proved a high sensitivity as shown by a panel of different agonists. Taken together, careful determination of possible bacterial contaminants allowed us to conclude that they are not the cause for macrophage activation by unloaded µRs in intact and disintegrated form.

Silica nanoparticles are sensed by the NLRP3 inflammasome and described to induce IL-1β release and caspase-1 activation NLRP3-dependently, as shown with THP-1 cells, human PBMCs, and murine BMMs [52,53,54]. In contrast to silica crystals, amorphous silica nanoparticles are described to be more biocompatible, but their possible adverse health effects are also discussed [55]. Effects of silica nanoparticles are dependent on their physicochemical properties, e.g., crystallinity, size, shape, and surface area [54]. Mainly nanoparticles, but not micro-sized particles, have been reported to trigger inflammation in BMMs [56].

We undertook a careful characterisation of the µRs used for TAM polarisation including their effect on cell viability and a possible role of NLRP3 inflammasome activation by unloaded µRs. Surprisingly, concentrations as low as 0.5 µR per cell reduced cell viability of HMDMs significantly. In contrast, a lowered viability was not detectable in the reporter cells, which also comprised THP-1 cells, in such low concentrations. As we reported previously, we did not observe a significant reduction in THP-1 viability in µR concentrations up to 100 µg/mL as determined by MTT assay [25]. The aim of the study was repolarization of TAMs, not killing them. Such approaches, i.e., depletion of M2-like TAMs (in contrast to M1-like TAMs) is another strategy for lung cancer therapy [5,57].

In line with inflammasome activation, IL-1β secretion was induced by µRs in HMDMs and BMMs. Data from *Nlrp3* KO BMMs supported that this IL-1β as well as cell viability is at least partially dependent on NLRP3. This effect was partly abrogated by particle disintegration, pointing to a role of shape in inflammasome activation by µRs. NLRP3 inflammasome activation occurs either by a two-step activation (canonical), a one-step activation, or by cytosolic LPS [40,58]. Crystals or particulate matter are described to act as a first and/or second stimulus in NLRP3 activation in macrophages [53,59,60]. 

Taken together, our data suggest the suitability of µRs to deliver poly(I:C) to macrophages. However, unloaded µRs induce a rather strong inflammatory activation themselves and there is a possible induction of cell death. In further steps, a detailed analysis including different µR materials and shapes, and in different macrophage polarization states is needed to further develop this promising tool for targeting and repolarizing TAMs in lung cancer.

In conclusion, silica µRs are a suitable tool for (i) targeting and (ii) delivery of poly (I:C) to TAMs. Both result in a desirable change of phenotype in TAMs. We provide data which show the urgent need for careful particle characterization in human macrophage targeting studies concerning possible contamination, stability, and material toxicity. Furthermore, by excluding THP-1 as a suitable model for TAMs, we demonstrate that the macrophage model employed for targeting should be chosen carefully.

## Figures and Tables

**Table 1:** HMDM cell culture specifications.

Type of Analysis	Plate Format	Seeding Density [Cells/Well]	Differentiation + Polarization [d]	Treatment Time [h]
IL1-β secretion	96-well	20,000	5	24
MTT-assay	96-well	50,000	5	24
flow cytometry (µR uptake)	24-well	250,000	5 + 2	1/3
flow cytometry (membrane expression)	12-well	244,000	5 + 1	48
Microscopy	24-well	200,000	5 + 2	1/3
live-cell microscopy (µR uptake)	96-well	40,000	5 + 2	1/3
live-cell microscopy (cytotoxicity)	96-well	40,000	5	24
mRNA expression	6-well	600,000	5 + 1	4
mRNA expression (TAM models)	12-well	500,000	6 + 1	/

**Table 2:** Conversion of µR concentrations in 96-well plates.

µR [µg/mL]	100	200	400	600	800
Treatment [µRs/cell]	0.5	1	2	3	4

**Table 3:** qPCR primer sequences.

Gene *	Accession Number	Forward (5′-3′)	Reverse (5′-3′)
*RNA18S5*	NR_003286.2	AGGTCTGTGATGCCCTTAGA	GAATGGGGTTCAACGGGTTA
*VEGFA*	NM_001171623.1	CGCTTACTCTCACCTGCTTCTG	GGTCAACCACTCACACACACAC
*HIF1A*	NM_181054.3	CGGGGACCGATTCACCAT	TTTCGACGTTCAGAACTTATCTTTT
*ABCA1*	NM_005502.4	CATCTGGTTCTATGCCCGCT	TCTGCATTCCACCTGACAGC
*ABCG1*	NM_016818.3	GCGCCAAACTCTTCGAGCTG	CGGATGCAACCTCCATGACAAA
*IL8*	NM_000584.4	GAGAAGTTTTTGAAGAGGGCTGA	GCTTGAAGTTTCACTGGCATCT
*CCL2*	NM_002982.3	TTGATGTTTTAAGTTTATCTTTCATGG	CAGGGGTAGAACTGTGGTTCA
*TNF*	NM_000594.4	CTCCACCCATGTGCTCCTCA	CTCTGGCAGGGGCTCTTGAT
*CXCL10*	NM_001565.4	GAGCCTACAGCAGAGGAACC	AAGGCAGCAAATCAGAATCG

* *VEGFA*: Vascular Endothelial Growth Factor A; *HIF1A*: Hypoxia Inducible Factor 1 Subunit Alpha; *ABCA1*: ATP Binding Cassette Subfamily A Member 1; *ABCG1*: ATP Binding Cassette Subfamily G Member 1; *IL8*: Interleukin 8; *CCL2*: C-C Motif Chemokine Ligand 2; *TNF*: Tumour Necrosis Factor; *CXCL10*: C-X-C Motif Chemokine Ligand 10.

**Table 4:** Receptor expression in reporter cell lines.

Reporter Cell Line	Receptor Expression
THP1-XBlue™	TLR1/2, TLR2/6, TLR4, TLR5, TLR8, NOD1, NOD2
HEK-Blue™-hTLR2	TLR1/2, TLR2/6, TLR3, TLR5, NOD1
HEK-Dual™ hTLR2 (NF/IL8) cells	TLR1/2, TLR2/6, NOD1

## Data Availability

The data presented in this study are available in the article and in the Supplementary Materials.

## Supplementary Materials

The following supporting information can be downloaded at: https://www.mdpi.com/article/10.3390/pharmaceutics15071895/s1, Figure S1: Flow cytometric gating strategy for µR uptake analysis. Figure S2: Flow cytometric gating strategy for HMDM surface markers in (M0). Figure S3: Reporter cell viability determined via MTT assay after 24 h. Figure S4: HEK-Blue™ IL1R reporter cell viability determined via MTT assay. Figure S5: Characteristics of poly(I:C) loaded µRs.
