# Supplementary material for: Nanostructured Microparticles Repolarize Macrophages and Induce Cell Death in an In Vitro Model of Tumour-Associated Macrophages
Source: Pharmaceutics. 2023 Jul 5;15(7):1895. doi: 10.3390/pharmaceutics15071895 (PMC10385046; doi:10.3390/pharmaceutics15071895)
Supplement: Supplementary file 1 [file pharmaceutics-15-01895-s001.zip › Figure S1 Al-Fityan et al.pdf]

Nanostructured Microparticles Repolarize Macrophages and Induce Cell Death in an In Vitro Model of Tumour-Associated Macrophages

Supplemental Figure S1

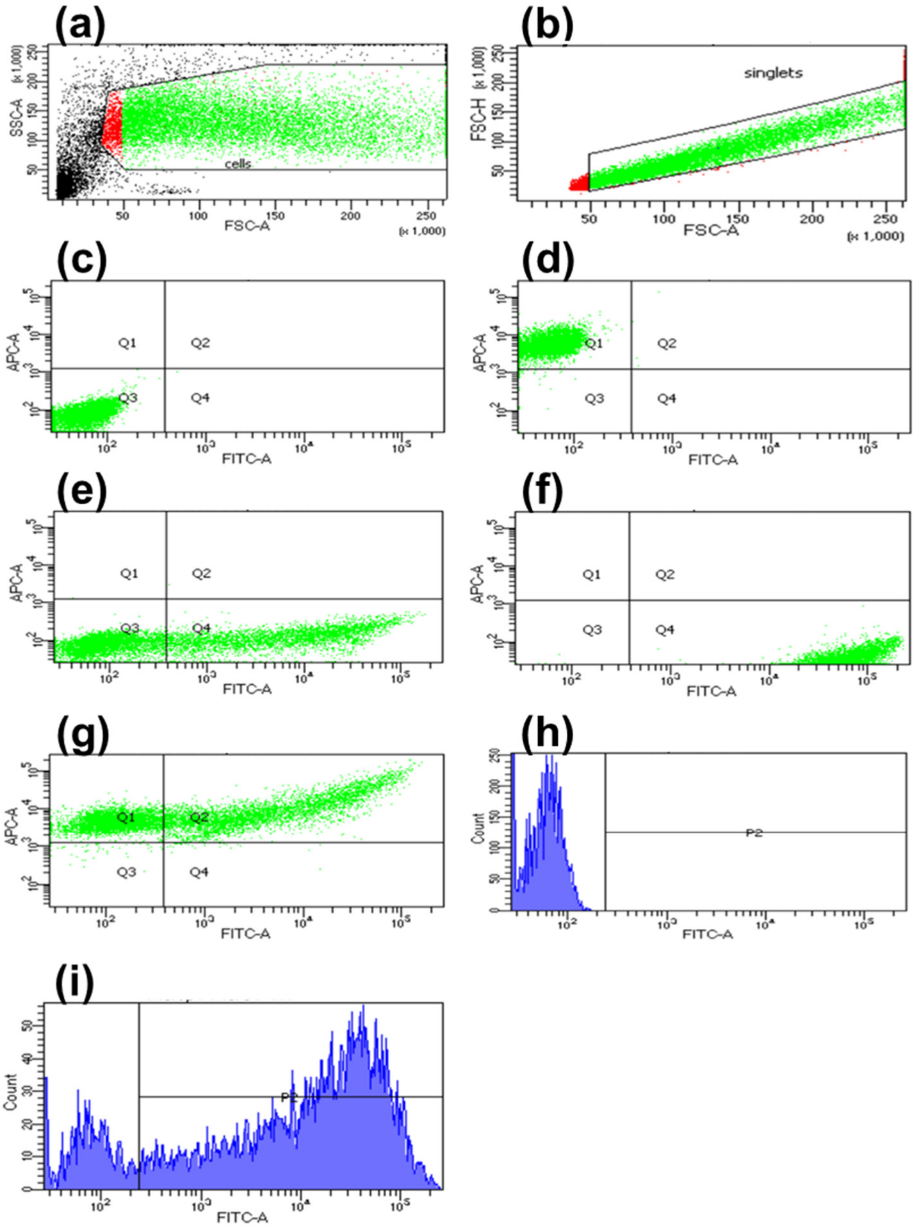

Figure S1. Flow cytometric gating strategy for µR uptake analysis. HMDMs (M0) were incubated with green fluorescent µRs (2 µRs/ cell, FITC) and stained with CellTracker™ Deep Red Dye (APC). (**a**) Events in FSC-A *vs*. SSC-A. (**b**) Singlets in FSC-H *vs*. FSC-A. (**c**) Unstained cells in APC-A *vs*. FITC-A (Q3). (**d**) Stained cells (Q1). (**e**) Unstained cells containing µRs (Q4). (**f**) µRs (Q4). (**g**) Stained cells containing µRs (Q2), Q1 and Q2 were used for evaluation. Representative histogram of cells without µRs (**h**) and with µRs (**i**). P2: FITC-positive events.
