# Supplementary material for: Nanostructured Microparticles Repolarize Macrophages and Induce Cell Death in an In Vitro Model of Tumour-Associated Macrophages
Source: Pharmaceutics. 2023 Jul 5;15(7):1895. doi: 10.3390/pharmaceutics15071895 (PMC10385046; doi:10.3390/pharmaceutics15071895)
Supplement: Supplementary file 1 [file pharmaceutics-15-01895-s001.zip › Figure S2 Al-Fityan et al.pdf]

Nanostructured Microparticles Repolarize Macrophages and Induce Cell Death in an In Vitro Model of Tumour-Associated Macrophages

Supplemental Figure S2

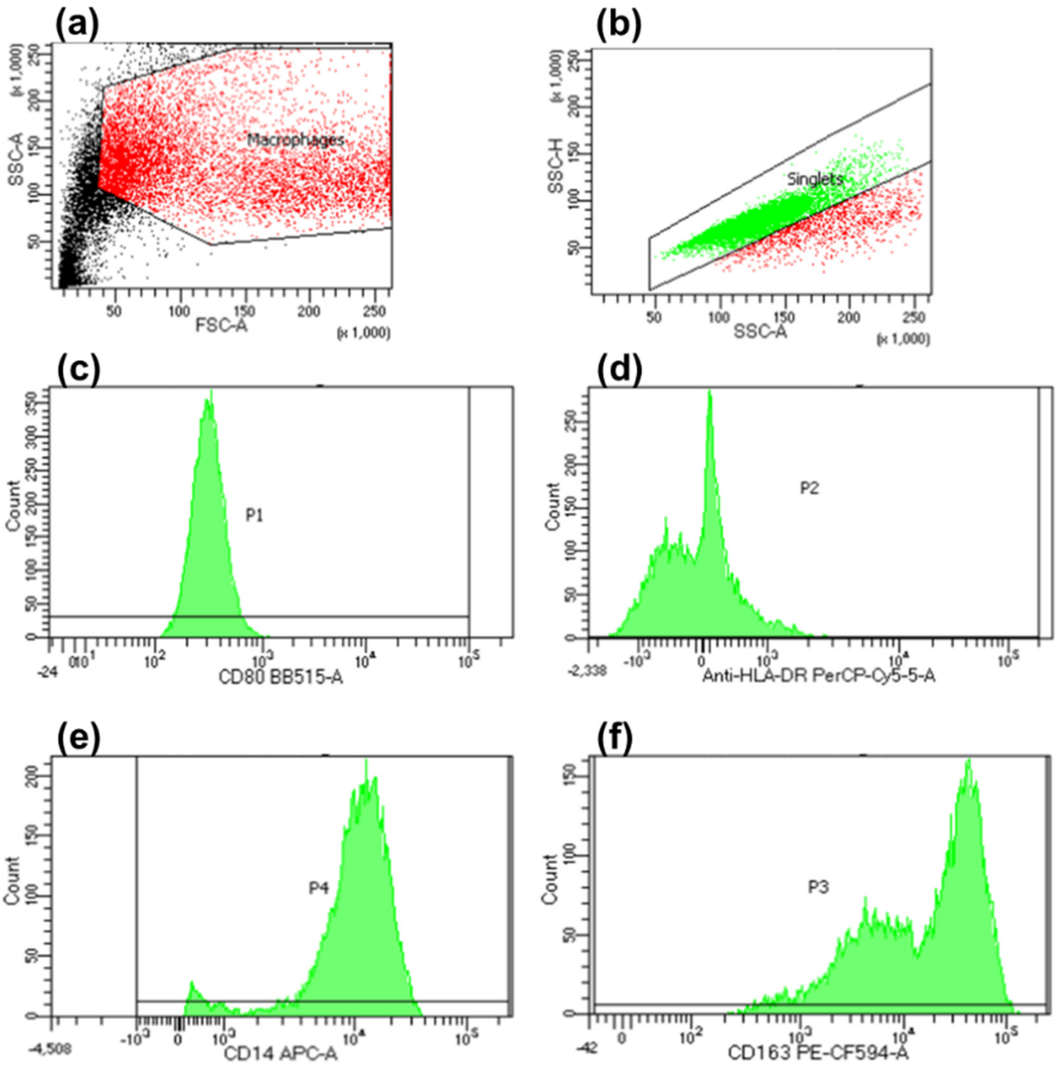

Figure S2. Flow cytometric gating strategy for HMDM surface markers in (M0) (**a**) Events in FSC-A vs. SSC-A. (**b**) Singlets in SSC-H vs. SSC-A. Representative histograms of surface marker expression (**c**) CD80, (**d**) HLA-DR, (**e**) CD14, and (**f**) CD163. P1-4: positive events.
