# Supplementary material for: Nanostructured Microparticles Repolarize Macrophages and Induce Cell Death in an In Vitro Model of Tumour-Associated Macrophages
Source: Pharmaceutics. 2023 Jul 5;15(7):1895. doi: 10.3390/pharmaceutics15071895 (PMC10385046; doi:10.3390/pharmaceutics15071895)
Supplement: Supplementary file 1 [file pharmaceutics-15-01895-s001.zip › Figure S3 Al-Fityan et al.pdf]

Nanostructured Microparticles Repolarize Macrophages and Induce Cell Death in an In Vitro Model of Tumour-Associated Macrophages

Supplemental Figure S3

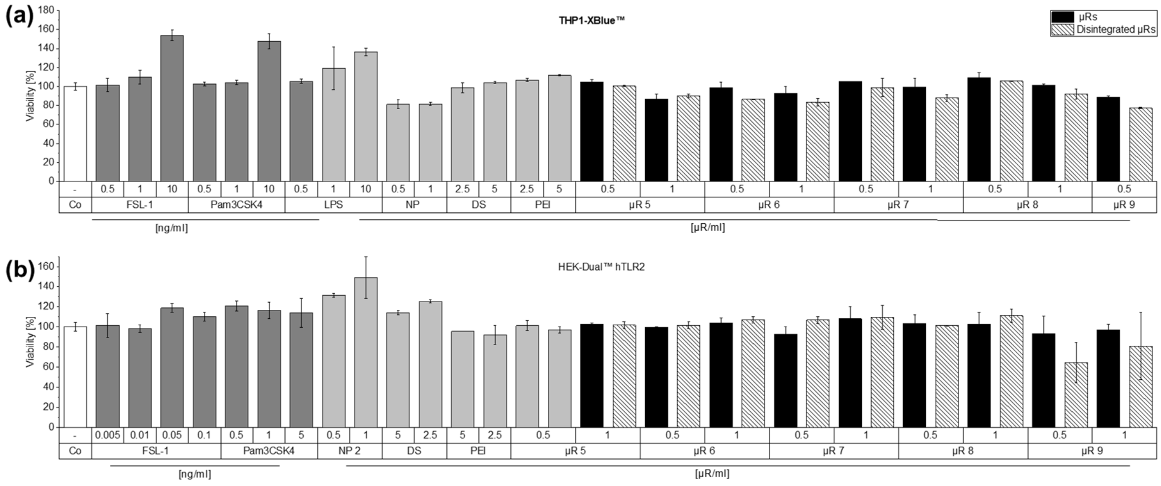

3

Figure S3. Reporter cell viability determined via MTT-assay after 24 h. (**a**): THP1-XBlue™ and (**b**) HEK-Dual™ hTLR2 cells, mean absorption normalized to untreated controls (Co, 100%). Cells were left untreated (Co), or incubated with FSL-1, Pam_3CSK_4, or LPS (ng/ml); components of silica µRs in an amount, which corresponds to 0.5 or 1 µR/cell. Silica nanoparticles (NP): 100 µg/ml, 200 µg/ml; dextran sulphate (DS) and branched polyethyleneimine (PEI): 2.5 and 5 µg/ml; with 100 or 200 µg/ml µRs (0.5 and 1 µR/cell) of different production batches (µR 5-9). µRs were unchanged (black) or disintegrated (shaded); *n* = 1, duplicates.
