# Supplementary material for: Nanostructured Microparticles Repolarize Macrophages and Induce Cell Death in an In Vitro Model of Tumour-Associated Macrophages
Source: Pharmaceutics. 2023 Jul 5;15(7):1895. doi: 10.3390/pharmaceutics15071895 (PMC10385046; doi:10.3390/pharmaceutics15071895)
Supplement: Supplementary file 1 [file pharmaceutics-15-01895-s001.zip › Figure S4 Al-Fityan et al.pdf]

Nanostructured Microparticles Repolarize Macrophages and Induce Cell Death in an In Vitro Model of Tumour-Associated Macrophages

Supplemental Figure S4

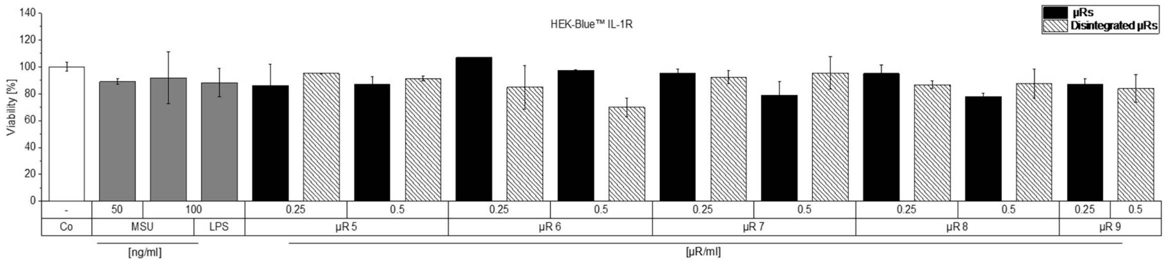

Figure S4: HEK-Blue™ IL1R reporter cell viability determined via MTT-assay. Cells were left untreated (Co) or incubated with positive controls (LPS (100 ng/mL) for 18 h ± monosodium urate crystals (50 and 100 µg/mL) for 6 h) or 50/100 µg/mL µRs (0.25 and 0.5 µR/cell) of different production batches (µR 5–9). µRs were unchanged (black) or disintegrated (shaded); duplicates.
