# Supplementary material for: Nanostructured Microparticles Repolarize Macrophages and Induce Cell Death in an In Vitro Model of Tumour-Associated Macrophages
Source: Pharmaceutics. 2023 Jul 5;15(7):1895. doi: 10.3390/pharmaceutics15071895 (PMC10385046; doi:10.3390/pharmaceutics15071895)
Supplement: Supplementary file 1 [file pharmaceutics-15-01895-s001.zip › Figure S5 Al-Fityan et al.pdf]

Nanostructured Microparticles Repolarize Macrophages and Induce Cell Death in an In Vitro Model of Tumour-Associated Macrophages

Supplemental Figure S5

Morphology analysis by Scanning Electron Microscopy

In order to characterize the manufactured formulations and the aspherical microparticles with respect to their morphology, scanning electron microscopy was used according to [1,2]. For this analysis we applied a Zeiss Evo HD 15 Electron Microscope (Carl Zeiss AG, Jena, Germany) equipped with a Lanthanum hexaboride (LaB6) cathode. The sample to be analysed was placed as a powder onto a silica wafer and then coated with a gold layer about 10 nm thick to render the surface conductive, avoiding the accumulation of electrons. To generate the thin gold layer, a Quorum Q150R ES sputter coater (Quorum Technologies Ltd., East Grinstead, UK) was used. The images were taken at a magnification of 10 and 1 kX at 5 kV (Figure S5; a, b).

1. Fischer, T.; Winter, I.; Drumm, R.; Schneider, M. Cylindrical Microparticles Composed of Mesoporous Silica Nanoparticles for the Targeted Delivery of a Small Molecule and a Macromolecular Drug to the Lungs: Exemplified with Curcumin and siRNA. *Pharmaceutics* **2021**, *13*, 844, doi:10.3390/pharmaceutics13060844.
2. Fischer, T.; Tschernig, T.; Drews, F.; Brix, K.; Meier, C.; Simon, M.; Kautenburger, R.; Schneider, M. siRNA delivery to macrophages using aspherical, nanostructured microparticles as delivery system for pulmonary administration. *European Journal of Pharmaceutics and Biopharmaceutics* **2021**, *158*, 284-293, doi:10.1016/j.ejpb.2020.11.024.

(a) 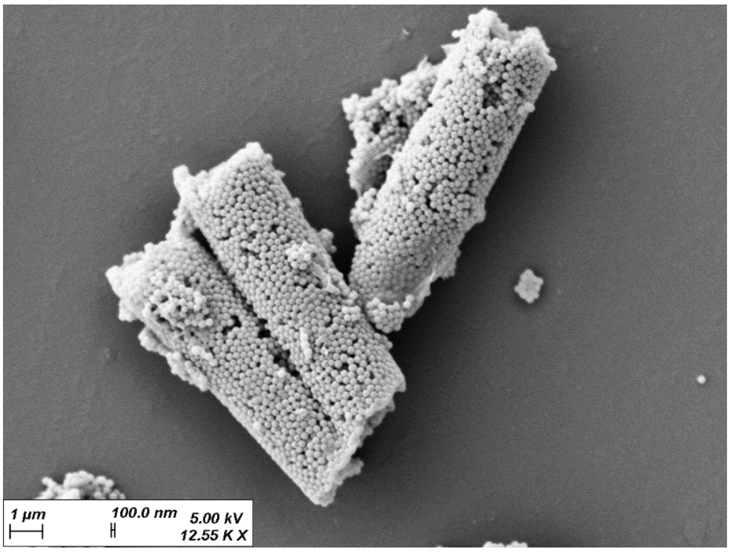

(b) 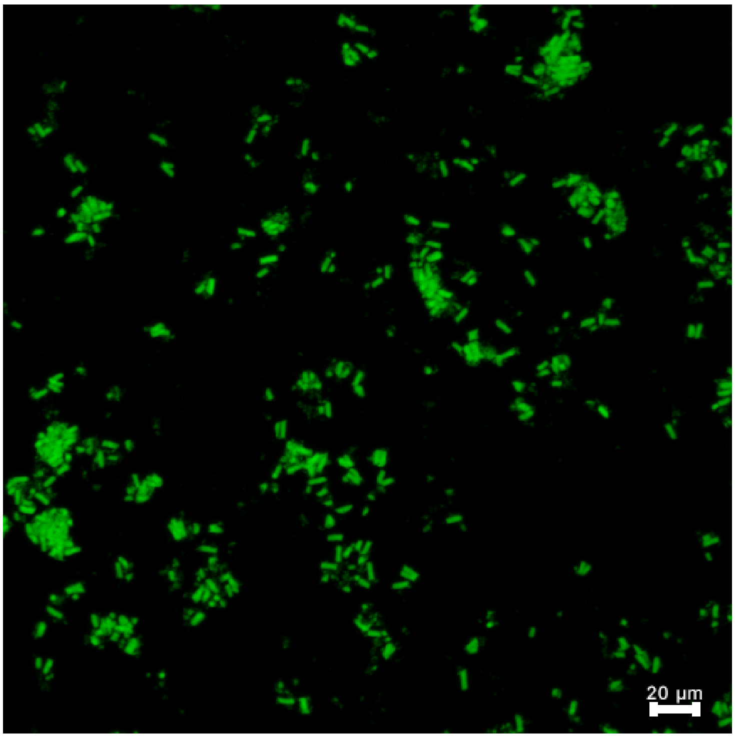

Figure S5: Characteristics of poly(I:C)-loaded µRs. (**a, b**) SEM images. The hierarchical structure of single SiO_2 NPs interconnected by the polymer coating can be observed. The cylindrical shape with 3 µm in diameter and 10 µm in length represents the dimensions of the template pores (see e.g. in Möhwald et al., 2017). The visualization was performed by an SEM setting after deposition of microrods from an aqueous microrod suspension. The rods remained well dispersed. (**c**) Cylindrical shape and low polydispersity of a representative batch of FITC-labelled, poly(I:C)-loaded µRs as shown by CLSM.
